# Creativity—the unconscious foundations of the incubation period

**DOI:** 10.3389/fnhum.2014.00215

**Published:** 2014-04-11

**Authors:** Simone M. Ritter, Ap Dijksterhuis

**Affiliations:** Behavioural Science Institute, Radboud University NijmegenNijmegen, Netherlands

**Keywords:** creativity, problem solving, incubation, mind wandering, sleep, unconscious processes

## Abstract

Creativity is one of the most important assets we have to navigate through the fast changing world of the 21st century. Anecdotal accounts of creative individuals suggest that oftentimes, creative discoveries result from a process whereby initial conscious thought is followed by a period during which one refrains from task-related conscious thought. For example, one may spend an embarrassing amount of time thinking about a problem when the solution suddenly pops into consciousness while taking a shower. Not only creative individuals but also traditional theories of creativity have put a lot of emphasis on this incubation stage in creative thinking. The aim of the present article is twofold. First, an overview of the domain of incubation and creativity is provided by reviewing and discussing studies on incubation, mind-wandering, and sleep. Second, the causes of incubation effects are discussed. Previously, little attention has been paid to the causes of incubation effects and most findings do not really speak to whether the effects should be explained by unconscious processes or merely by consequences of a period of distraction. In the latter case, there is no need to assume active unconscious processes. The findings discussed in the current article support the idea that it is not merely the absence of conscious thought that drives incubation effects, but that during an incubation period unconscious processes contribute to creative thinking. Finally, practical implications and directions for future research will be discussed.

Important achievements in the arts and sciences depend on creativity (Feist and Gorman, 1998; Kaufman, 2002), and creativity is associated with the development of new social institutions (Bass, 1990; Mumford, 2002) and economic growth (Amabile, 1997; Simonton, 1999). It is generally accepted that a creative idea or a creative solution to a problem has to be *novel* (i.e., original) and *useful* (Amabile, 1983; Runco and Pritzker, 1999). Creativity is not limited to the realms of greatness, but can also be found in daily life, for example, when one has to accomplish a task in a new way (Cropley, 1990) or when one has to adapt to changes (Runco, 2004). Today’s world of continuous change thrives on creative individuals.

Creativity has been related to cognitive abilities, expertise, and practice (Patrick, 1986; Amabile, 1996; Runco, 2004; Ericsson, 2006; Sawyer, 2012), and one may expect that creativity mainly thrives on extensive conscious thought. However, creative individuals, in describing their work habits or the process of creative problem solving, have suggested that oftentimes, creative ideas result from a period of incubation—a process whereby initial conscious thought is followed by a period during which one refrains from task-related conscious thought (for anecdotal accounts, see Ghiselin, 1952). The most frequently cited anecdote is probably the one from the mathematician Poincaré:

“[…] I left Caen, where I was living, to go on a geologic excursion under the auspices of the School of Mines. The incidents of the travel made me forget my mathematical work. Having reached Coutances, we entered an omnibus to go some place or other. At the moment when I put my foot on the step, the idea came to me, without anything in my former thoughts seeming to have paved the way for it, that the transformations I had used to define the Fuchsian functions were identical with those of non-Euclidian geometry. I did not verify the idea; I should not have had the time, as, upon taking my seat in the omnibus, I went on with a conversation already commenced, but I felt a perfect certainty. On my return to Caen, for conscience’ sake, I verified the result at my leisure.” (Poincaré quoted in Hadamard, 1945, p. 13).

In addition, several famous anecdotes suggest that sleep facilitates creativity, ranging from musical compositions to scientific insights (Mazzarello, 2000). In speaking of the attainment of solutions, “Beatle” Paul McCartney announced that he came up with the melody for “Yesterday” in a dream, and the Nobel Prize winner Loewi woke up with the idea for how to experimentally prove his theory of chemical neurotransmission.

The idea that a period of incubation might facilitate creativity has not only been suggested by creative minds, but has also been stressed in creativity models. Wallas (1926) proposed that the creative process entails four stages: Preparation (acquisition of knowledge to some task), Incubation (process that occurs when conscious attention is diverted away from the task), Illumination (creative idea flashes into sight), and Verification (creative idea is subjected to evaluation). Certainly a creative idea may be found *before* a decrease in conscious effort, that is, before the Incubation stage. Sometimes, however, a period of incubation seems to precede creative breakthroughs as illustrated above for several scientific discoveries and artistic compositions.

Sparked by the anecdotal accounts on incubation and creativity, various attempts have been made to investigate incubation effects. As demonstrated by a Google Scholar search (Sio and Ormerod, 2009), with the search restricted to the years 1997–2007 and the subject areas to social sciences, arts, and humanities, the term *incubation* along with either *creativity, insight*, or *problem*, yielded more than 5000 articles. Empirical research has shown that a period of incubation indeed helps creativity (Dodds et al., 2003; Sio and Ormerod, 2009).

However, it is not yet clear why incubation is helpful. The moderators discovered thus far do not really speak to whether the effects should be explained by unconscious processes or merely by other consequences of a period of distraction (e.g., relaxation, forgetting of fixating elements, mental set-shifting) without the need to assume active unconscious processes (see also Orlet, 2008). The aim of the current article is to provide an overview of the domain of incubation and creativity, and to review and discuss findings that speak to whether during an incubation period unconscious processes contribute to creative thinking, or whether it is merely the absence of conscious thought that drives creativity. Finally, practical implications and directions for future research will be discussed.

## Incubation

Many anecdotal accounts and traditional theories of creativity have put emphasis on incubation. The basic phenomenon is a familiar one: we are working on a problem, we can’t solve the task, we leave it aside for some period of time—the incubation period—and when we return attention to the task we have some new insight that helps us to solve the problem. In general, there are two frequently used methods to conduct incubation experiments. In the “interpolated activity” method, participants work on a task or problem for a period of time, are then given an incubation period, and finally return to the task. Participants’ performance is compared with that of a control group of people who worked on the same problem continuously. In the “multiple trial or multiple item” method, multiple tasks or problems are presented and, afterwards, items that have not been solved are re-administered. For example, participants work for one minute apiece on several problems. Then, the unsolved problems are re-administered for one minute apiece. It is presumed that the time between the first and second encounter with the tasks or problems allows incubation to occur. Some researchers using this approach also insert an incubation period between the first and second encounter with the problems. For example, after the first encounter with the problems participants perform a distractor task and after this incubation interval return to work for a certain time on problems that they did not solve. Participants’ performance is compared with that of a control group, and if a within-participant design is used, the increase in number of problems is used.

Whereas some studies reported strong incubation effects (e.g., Kaplan, 1989; Smith and Blankenship, 1989; Smith and Dodds, 1999; Dodds et al., 2003), others have failed to find any effects at all (e.g., Olton and Johnson, 1976). To resolve the uncertainties surrounding incubation effects, Dodds et al. (2003) conducted a review of experimental literature on incubation in problem solving and creativity, and revealed that 29 out of 39 experiments have found a significant effect of incubation. The authors suggested that incubation length and preparatory activities can increase incubation effects. Moreover, the authors demonstrated that presenting a clue during the incubation period can either have strong positive (if the clue is useful) or negative effects (if the clue is misleading). For example, “ocean” or “floor” could be a misleading clue when trying to find a fourth word that functions as an associative link between the three items “ship, outer, crawl”, whereas “space” could be a useful clue. Sio and Ormerod (2009) conducted a statistical meta-analytic review of empirical studies of incubation. In their meta-analysis 117 independent studies were included, and the contributions of moderators such as problem type, presence of cues, and lengths of preparation and incubation periods were investigated. Overall, a positive incubation effect was found. In a recent study, Gilhooly et al. (2013) investigated interactions between the type of creativity task (verbal or spatial) and the type of incubation activity (verbal or spatial) on creative performance. Experimental groups, after 5 min of conscious work on a verbal creativity task (Alternative Uses Task) or a spatial creativity task (Mental Synthesis), had a 5-min incubation period that involved either spatial (Mental Rotation) or verbal (Anagrams) tasks. Following incubation, participants resumed their main task for a further 5 min. Control groups undertook Alternative Uses or Mental Synthesis for 10 min without any incubation periods. Significant incubation effects were found overall and there were interactions in that spatial incubation benefited verbal fluency and verbal-rated creativity, and verbal incubation benefited spatial-task fluency and spatial-rated creativity but not vice versa. These findings suggest that an interpolated incubation activity of a dissimilar nature to the target task leads to stronger effects of incubation as compared to an interpolated activity similar to the target task.

Not only the task that is performed during an incubation period, but also the time interval of an incubation period can vary. It can vary from a few moments or a night of sleep through days or weeks away from the problem. An example of a relatively short incubation period is *mind-wandering—*a state of mind that occurs spontaneously, and largely autonomously, whenever an awake individual is not engaged in a cognitively demanding task. Research on mind-wandering has a long history, and was recently popularized by Smallwood et al. (2003) who used thought sampling and questionnaires to investigate mind-wandering. In past and recent literature, alternative names to the term mind-wandering (Smallwood and Schooler, 2006; Mason et al., 2007) have been used, such as “day dreaming” (Giambra, 1979), “spontaneous thought” (Christoff et al., 2011), “task-unrelated thought” (Giambra and Grodsky, 1989; Smallwood et al., 2003), and “stimulus independent thought” (Teasdale et al., 1995). In a recent study, Baird et al. (2012) examined whether creative performance was facilitated differentially by engaging in mind-wandering (i.e., a 0-back task, an undemanding task without memory load that has been shown to elicit mind-wandering, Smallwood et al., 2009), a demanding task (i.e., a 1-back working memory task), a rest period, or no break between creativity problems. To measure creative performance, the Unusual Uses Task (a task that requires participants to generate as many unusual uses as possible for a common object) was used. All participants performed two Unusual Uses Task problems (2 min per problem) to measure baseline creative performance. Subsequently, participants were assigned to one of the four between-subjects conditions. After the incubation interval (or following the baseline measure, in the case of the no-break condition), participants worked on the Unusual Uses Task again. Four problems (2 min per problem) were presented in a random order: two problems that were identical to the problems presented at baseline and two new problems. Engaging in an undemanding task during an incubation period led to significant increases in creative solutions to the target problems as compared to the demanding task, rest, and no break conditions. This improvement was observed only for repeated-exposure problems, which demonstrates that it resulted from an incubation process rather than a general increase in creative problem solving. The unrelated thoughts that occur during mind wandering uniquely seem to facilitate incubation. According to Baird et al., one possible explanation may be that mind wandering enhances creativity by increasing unconscious associative processing, as predicted by the spreading-activation account of incubation (e.g., Yaniv and Meyer, 1987; Dijksterhuis and Meurs, 2006).

In recent years, functional Magnetic Resonance Imaging (fMRI) research has been used to focus on understanding how the brain generates the spontaneous and relatively unconstrained thoughts that are experienced when the mind wanders. One candidate neural mechanism for mind-wandering is a network of regions in the frontal and parietal cortex known as the default mode network (Mason et al., 2007; Christoff et al., 2009). The default mode network, also called the default network, default state network, or task-negative network, is defined as a set of interconnected brain regions including the medial prefrontal cortex (MPFC), posterior cingulate cortex (PCC), and lateral and medial temporal lobes (Spreng et al., 2010). It is a brain system that is especially active when an individual is not focused on the outside world (Buckner et al., 2008) and when cognitive control is low (Andreasen, 1995). Moreover, it has been related to complex, evaluative and unconscious forms of information processing (Vincent et al., 2007; Yang et al., 2010), and it contrasts with the cognitive control network (Fox et al., 2005)—a set of brain regions including the anterior cingulate cortex (ACC), dorsolateral prefrontal cortex (DLPFC), inferior frontal junction (IFJ), anterior insular cortex (AIC), dorsal pre-motor cortex (dPMC), and posterior parietal cortex (PPC; Cole and Schneider, 2007). Indeed, when one network is activated, the other is deactivated (Fox et al., 2005). In addition to these findings, structural MRI research has provided a first indication that the default mode network may be involved in creativity. Jung et al. (2010) have linked cortical thickness measures to psychometric measures of creativity and found a negative correlation between creative performance and activity in the lingual gyrus and a positive correlation between creative performance and grey matter volume in the right PCC, a brain area that is part of the default mode network.

In a recent structural MRI study, Kühn et al. (2013) provided further support for the involvement of the default mode network in creativity. Participants performed a well-established creativity task by which a participant’s cognitive flexibility and the average uniqueness and average creativity of a participant’s ideas were assessed. For all psychometric measures of creativity a positive correlation was observed between inter-individual differences in creative performance and inter-individual differences in volume of the default mode network. Based on these findings, it can be assumed that greater volume in the default mode network (i.e., in the counterpart of the cognitive control network) provides more neural resources for generating creative ideas. These findings suggest that less controlled processes such as mind-wandering are important in creativity. One relatively controversial finding is that periods of mind-wandering are associated with increased activation in both the default and executive system, a result that implies that mind-wandering may often be goal oriented (Smallwood and Schooler, 2006; Smallwood et al., 2009). Apart from studies about the default mode network, there are several important other studies on neuroimaging and creativity. For example, the research from Reverberi et al. (2005) demonstrates that the lateral frontal cortex impairs problem solving, and the research by Kounios and Jung-Beeman (2009) on the cognitive neuroscience of insight suggests that insight is the culmination of a series of brain states and processes operating at different time scales. Recently, Dietrich and Kanso (2010) reviewed 72 neuroimaging studies on creativity and insight and concluded that the neuroscientific literature on creativity, thus far, is self-contradicting and that creative thinking does not appear to critically depend on any single mental process or brain region. The default mode network can, therefore, be considered one, but not the single neural underpinning of creativity.

Whereas mind-wandering can be considered a relatively short incubation period, sleep can be considered an incubation period that covers a longer period of time. Sleep is divided into two broad types, rapid eye movement (REM) sleep and non-rapid eye movement (NREM) sleep. Each type has a distinct set of associated physiological and neurological features. REM sleep is a stage of sleep characterized by the rapid and random movement of the eyes, and typically occupies 20–25% of total sleep. During REM, the activity of the brain’s neurons is quite similar to that during waking hours and subjects’ vividly recalled dreams mostly occur during REM sleep. Unlike REM sleep, during NREM sleep there is usually little or no eye movement and dreaming is rare. The differences in the REM and NREM activity reported is believed to arise from differences in the memory stages that happen during the two methods of sleep (Manni, 2005). For example, Stickgold et al. (1999) have shown that cognition during REM sleep is qualitatively different from that of waking and NREM sleep, and may reflect a shift in associative memory systems. They suggest that this shift in cognitive processing is responsible, in large part, for the bizarre nature of dreams and may serve to enhance the strength of associations between weakly associated memories, an important skill underlying creative thinking. The mental activity that takes place during NREM sleep is believed to be thought-like, whereas REM sleep includes hallucinatory and bizarre content (Manni, 2005). Thus far, sleep research has mainly been focused on memory performance. A prominent finding is that sleep, and certain stages of sleep in particular, are important in memory processing, resulting in delayed learning without the need for further practice or task engagement (Stickgold et al., 2001). These findings of sleep-dependent learning are now strongly supported by cellular and molecular evidence of sleep-dependent plasticity across a broad range of phylogeny (Bennington and Frank, 2003). Yet memory consolidation is only one of many cognitive virtues possessed by the human brain, another is creativity.

The link between creativity and sleep, especially dreaming, has long been a topic of intense speculation (Stickgold and Walker, 2004). In recent years, the facilitatory effect of sleep on creativity has also received empirical support. Research from Barrett (1993) has shown that college students incubated answers to real-life homework and other objective problems on which they were working, finding that in one week’s time, half of the students had dreamed about their topic and 25% had a dream that provided an answer. Barrett (2001) also interviewed modern artists and scientists (including Nobel Prizes winners) about their use of their dreams and concluded that while anything—math, musical composition, business dilemmas—may get solved during dreaming, the two areas dreams are especially likely to help are anything where vivid visualization contributes to the solution and any problem where the solution lies in thinking outside the box—i.e., where the person is stuck because the conventional wisdom on how to approach the problem is wrong. Moreover, in an experimental study Wagner et al. (2004) have shown that sleep inspires creative insight. Subjects completed a number reduction task, and each numerical sequence could be completed in a slow, stepwise way, but the trials could also be completed according to a hidden, more abstract rule that would speed up participants’ responses. The initial training was followed by 8 h of nighttime sleep, nighttime wakefulness, or daytime wakefulness. Of the people who slept before they resumed, almost 60% discovered the rule, as opposed to 23% of the people in the two groups that did not sleep. Thus, participants who got several hours of sleep were more than two times as likely during retesting to gain insight into a hidden rule built into the task.

In addition, sleep has been shown to enhance important aspects of creativity, including cognitive flexibility and the ability to find remote associations. In a study on cognitive flexibility across the sleep-wake cycle, Walker et al. (2002) found that when woken from REM sleep, participants had a 32% advantage in the number of anagrams solved compared with NREM awakenings, which were equal to that of wake time performance. These findings suggest that REM sleep may offer a different mode of problem solving compared with wake and NREM. The authors hypothesized that REM sleep is highly conducive to fluid reasoning and flexible thought due to the lack of aminergic dominance in REM sleep. In a study on the ability to find remote associations, Sio et al. (2012) participants were presented with a set of Remote Associates Test (RAT) items. Each RAT item contains a triplet of words presented horizontally along with a blank space. For each item, the participant has to find a fourth word that functions as an associative link between these three words (e.g., cookies, sixteen, heart: ………; the answer to this item is sweet: cookies are sweet, sweet sixteen, sweetheart). Reaching a solution requires creative thought as the first, most probable associate to each of the items is often not correct, so the participant must think of more remote associations (i.e., distantly related information) to connect the three words. In the current study the RAT items varied in difficulty as a function of the strength of the stimuli–answer associations. After a period of sleep, wake, or no delay, participants reattempted earlier unsolved problems. The sleep group solved a greater number of difficult RAT items than did the other groups, but no difference was found for easier RAT items. These findings suggest that sleep facilitates creative thinking for harder problems. While evidence for the role of sleep in creative problem-solving has been looked at by prior research, underlying mechanisms such as different stages of sleep had not been explored. Cai et al. (2009) used the RAT, and tested participants in the morning, and again in the afternoon, after either a nap with REM sleep, one without REM or a quiet rest period. Participants grouped by REM sleep, non-REM sleep and quiet rest were indistinguishable on measures of memory. Most importantly, although the quiet rest and NREM sleep groups received the same prior exposure to the task, they displayed no improvement on the RAT test, whereas the REM sleep group improved by almost 40% over their earlier performances. The authors hypothesized that the formation of associative networks from previously unassociated information in the brain, leading to creative problem-solving, is facilitated by changes to neurotransmitter systems during REM sleep. Thus, REM sleep is assumed to enhance the integration of unassociated information for creative problem solving.

To recap, various attempts have been made to investigate incubation effects in creativity and creative problem solving. The conclusion of the literature is that overall, a positive incubation effect can be observed. Especially the work by Dodds et al. (2003) and Sio and Ormerod (2009), who conducted reviews of empirical studies of incubation, justify the conclusion that incubation can enhance creative performance. This is also supported by research on mind-wandering and sleep, which can be seen as short and relatively long periods of incubation. However, the process(es) underlying incubation effects remain unclear. In the next section, we aim to shed light on the question whether during an incubation period unconscious processes contribute to creative thinking, or whether it is merely the absence of conscious thought that drives incubation effects.

## Mechanisms Underlying Incubation Effects

Whereas the effects of incubation are generally accepted (Sio and Ormerod, 2009), its causes are controversial. The main debate between different theories is about whether during an incubation period unconscious processes contribute to creative thinking (*unconscious work theory*), or whether it is merely the absence of conscious thought that drives creativity (*conscious work theory*). Historically, incubation effects refer to the idea that setting a problem aside for a while helps creative thought and problem solving as *unconscious processes* are working on the problem while the individual is not consciously thinking about the problem (see Wallas, 1926, as well as, e.g., Hadamard, 1945; Kris, 1952; Rugg, 1963; Kubie, 1985). That is, the unconscious actively thinks and contributes to solving a problem (see also Koestler, 1964; Claxton, 1997). In contrast, conscious work theories have ascribed incubation effects on creative performance to *relaxation* (being well-rested, one can do better the next time one engages in the problem; Helmholtz, 1896; Woodworth and Schlosberg, 1954) and to the effects of *facilitating cues* from the environment (environmental cues trigger retrieval of previously un-retrieved relevant information; e.g., Yaniv and Meyer, 1987; Langley and Jones, 1988). Moreover, sometimes old and inappropriate ideas can cause *mental fixation*, impeding the generation of new and appropriate ideas (Smith, 2003). Therefore, in addition to relaxation and facilitating cues, it has been suggested that incubation effects can lead to *forgetting of fixating elements* (Smith and Blankenship, 1989; Segal, 2004) and to *mental set-shifting* (wrong cues become less accessible, leading to a fresh, new and unbiased start; Schooler and Melcher, 1995).

Recently, Gupta et al. (2012) investigated whether high-frequency candidate answers should be avoided in order to find creative solutions in for instance a RAT. They tested individual differences in creativity as measured with a complex problem-solving task, and developed a computational model of the RAT. Findings showed that individuals performed poorly on the RAT when they were biased to consider high-frequency candidate answers. Storm and Angelo (2010) investigated whether inhibition may facilitate creative problem solving by providing a mechanism by which to bypass fixation. They measured participants’ retrieval-induced forgetting and, thereafter, participants had to solve RAT problems. Half of the participants were exposed to misleading associates prior to problem solving (fixation condition) and half were not (baseline condition). Correlating the retrieval-induced forgetting measure with performance on the RAT revealed that the propensity to inhibit irrelevant information comes at a price, as potentially relevant information may be inhibited. However, inhibition can also provide a means by which to overcome fixation and, thereby, facilitate creativity. There is no denying that a period of distraction allows for forgetting of fixation and/or mental set-shifting, relaxation, and exposure to environmental cues, and that these effects can contribute to creative thoughts or problem solving. However, it can be questioned whether these effects are the only benefit of an incubation period, or whether during an incubation period unconscious processes contribute to creative thinking.^1^

Research from Bowers et al. (1990) suggests that the unconscious is able to “close in” on the correct answer some time before the answer is accessible to consciousness. They asked participants to find a target word while they were given successive hints, such as an associated word. Individuals felt clueless for some time and then suddenly came up with the correct answer. However, analysing the prior guesses revealed that individuals were slowly getting closer to the right solution before the solution reached consciousness. Participants’ successive guesses, thus, converged towards the correct answer. Moreover, a study conducted by Betsch et al. (2001) demonstrated that people can unconsciously integrate large amounts of information. Participants watched TV ads shown on a computer screen and simultaneously the numerical increases and decreases of hypothetical shares were shown at the bottom of the screen. Participants could not correctly answer specific questions about the shares, but they had developed a liking or disliking towards each of the shares. These findings suggest that participants processed and integrated the information while they were attending to the TV ads. Recently, Ric and Muller (2012) have shown that people can unconsciously initiate and follow arithmetic rules, such as addition. In several studies participants were instructed to detect whether a symbol was a digit, and this symbol was preceded by two digits and a subliminal instruction (i.e., the “add” instruction or a control instruction). Participants were faster at identifying a symbol as a number when the symbol was equal to the sum of the two digits and they received the instruction to add the digits. In line with these findings, Sklar et al. (2012) demonstrated that presenting participants with additions or subtractions subliminally leads to higher accessibility of correct answers (i.e., answers could be verbalized faster) than incorrect answers. A recent review on unconscious higher-order cognition conducted by Van Gaal et al. (2012) revealed strong evidence for unconscious response-inhibition, conflict resolution, as well as for error detection. Importantly, they also concluded that people can unconsciously integrate multiple pieces of information across space and time.To resume, evidence from various research areas demonstrates that processes that we consider thought processes can ensue unconsciously. This makes it reasonable to assume that thought processes in the service of creativity and problem solving can, in principle at least, also take place unconsciously.

The idea that during an incubation period unconscious processes are active was one of the building blocks of Unconscious Thought Theory (Dijksterhuis and Nordgren, 2006). Unconscious thought, that is, “deliberation in the absence of conscious attention directed at the problem,” (Dijksterhuis et al., 2006, p.1005) has mainly been studied in the context of decision-making (Strick et al., 2011; see also Dijksterhuis and Nordgren, 2006; Bargh, 2011; Nieuwenstein and van Rijn, 2012). In the literature on unconscious thought in decision-making, participants are typically first presented with information pertaining to a decision. Thereafter, they are distracted for a while, before they make a decision. For example, Bos et al. (2008) compared participants decision performance after three conditions, a conscious thought condition and two incubation conditions, that is, an unconscious thought condition and a mere distraction condition. Whereas participants in the unconscious thought condition were told that they would engage in an unrelated task before returning to the actual task, participants in the mere distraction condition were told that they had finished the task and would move on to unrelated tasks. In the mere distraction condition participants were, thus, distracted just as in the unconscious thought condition, but did not have a problem-solving goal. A period of distraction only improved decision-making in the unconscious thought condition, that is, when participants expected to make a decision following the distraction period. Comparing an unconscious thought condition with a mere distraction condition provides evidence for true, active thought taking place unconsciously. Given the evidence for unconscious thought processes that we have from multiple research areas (e.g., decision making, lie detection), the question raises whether there is also evidence for unconscious thought effects in the domain of creativity.

Dijksterhuis and Meurs (2006) investigated the relation between different thought processes and the generation of creative ideas. In several experiments participants were asked to generate a list of items (new names for products, names of places beginning with a certain letter, things one can do with a brick), and three conditions were compared. In the immediate condition (i.e., the baseline condition) participants started right after receiving the instruction. In the conscious thought condition, participants were given three minutes to consciously think about the items before they were given time to list them. In the unconscious thought condition, people were first given the instruction, and were then distracted for three minutes before they were given the opportunity to list the items. Conscious thought led to more accessible items and to items in line with a cue, whereas unconscious thought led to more inaccessible items and to items diverging from the cue. Moreover, unconscious thought led to more creative and unusual items than conscious thought. In all experiments, unconscious thinkers also differed significantly from participants who were not given time to think at all. These findings suggest that whereas conscious thought may be focused and convergent, unconscious thought may be more associative and divergent. Ritter et al. (2012b) investigated the role of unconscious thought for both idea generation and idea selection. Participants generated creative ideas immediately, after conscious thought, or after a period of unconscious thought. After having listed their ideas, participants selected their most creative idea. Performance in idea generation was similar between conscious and unconscious thought; however, individuals who had unconsciously thought about ideas were better in selecting their most creative idea. These findings are in support of the idea that unconscious processes actively contribute to creativity, as it is unlikely that these findings are the consequence of set-shifting or relaxation. During task instruction no examples were provided and no hints or cues were given, meaning that no fixating elements or specific mental sets were induced that could have become less accessible, changed, or forgotten altogether during a period of distraction. Recovering from fatigue is also unlikely to account for the current findings, as incubation effects also occurred in the study when a cognitively demanding task (n-back task) was used as distracter task (Dijksterhuis and Meurs, 2006).

Zhong et al. (2008) investigated the effect of unconscious thought on the ability to find remote associations, as measured by the RAT. Participants were presented difficult RAT triads (selected from Bowden and Jung-Beeman, 2003). Afterwards, participants in the conscious thought condition were told that they had 5 min to think about these triads, and during this time, they were shown the screen containing all triads, but were not allowed to write down notes or answers. Participants in the unconscious-thought condition were told that they would engage in an unrelated task before returning to the word task. Participants in the distraction condition were told that they had finished the task and would move on to unrelated tasks. To prevent conscious thought about the RAT items in the unconscious thought and mere distraction conditions participants completed a 2-back task for 5 min (see Dijksterhuis (2004)). After 5 min of conscious thought or distraction, all participants engaged in a lexical decision task (Bargh et al., 1995). Strings of letters appeared on the center of the screen, and participants indicated whether or not each string constituted an English word by pressing one of two buttons. The letter strings included the RAT answers plus control words. After completing the lexical decision task, participants in all three conditions were again shown the RAT items and were asked to report their answers. In the current research two separate outcomes of the RAT test were assessed: implicit accessibility of correct RAT answers (i.e., mental accessibility of RAT answers, as measured by a lexical decision task) versus expression of those correct answers (Wegner and Smart, 1997). A period of incubation, compared with the same duration of conscious thought, did not increase the reporting of correct answers. The results on accessibility, however, revealed a striking difference: Unconscious thought, compared with conscious thought and mere distraction, increased the mental accessibility of RAT answers. These results are consistent with unconscious thought theory, which systematically differentiates conscious and unconscious thought processes, and suggest that unconscious processing is more adept at associating and integrating information than conscious processing is (Dijksterhuis and Nordgren, 2006). Importantly, in the unconscious-thought condition the level of activation of RAT answers was higher than in the mere-distraction condition, which suggests that the increased accessibility after unconscious thought was not due to relaxation, forgetting or the release of incorrect associations (i.e., “mental set-shifting”). These findings indicate that unconscious processes may actively facilitate the discovery of remote associations, an important mental skill underlying creative thinking, and may contribute to divergent thinking.

Yang et al. (2012) investigated under what conditions unconscious thought can outperform conscious thought on creativity tasks. Their results demonstrated that unconscious thought did not provide creative advantage over conscious thought when deliberation duration was either short or long (1 or 5 min, respectively). However, when deliberation duration was of a moderate length (3 min), the creative output of unconscious thought exceeded that of conscious thought. These findings suggest that the duration of unconscious thought has an inverted-U shaped relationship with creativity. However, as different tasks require different amounts of mental effort, the appropriate duration of a moderate length can be assumed to be task dependent. In line with these findings, a meta-analysis on unconscious thought effects on decision-making (Strick et al., 2011) has shown that unconscious thought effects are larger with moderate unconscious thought intervals. Moreover, unconscious thought effects in decision-making have been shown to be larger when a task is used that does not require much processing capacity, that is, a relatively undemanding task. Similarly, Sio and Ormerod’s (2009) statistical meta-analytic review of incubation effects revealed that the benefits of an incubation period are greater when participants are occupied by an undemanding task than when they engage in either a demanding task or no task at all (Sio and Ormerod, 2009). Moreover, Ellwood et al. (2009) demonstrated that the type of break during the incubation period effects later solutions. As the functional fixedness theory as well as the general fatigue theory predict that a break, independent of its content, should be equally effective in producing an incubation effect, these findings suggest that systematic effects beyond relief from functional fixedness or general fatigue are at play.

Gallate et al. (2012) and Ritter et al. (2012b) investigated whether one can manipulate unconscious thought processes. In the study from Gallate et al., participants were either aware or unaware that they would soon be returning to a divergent thinking task. During the break period, all participants were distracted from the task (they did an arithmetic task), ensuring that any ongoing problem solving was not conscious, but unconscious. Immediately after finishing the arithmetic task, participants returned to the divergent thinking task. Participants in the aware condition had significantly higher post-break creativity scores than those in the unaware condition. Ritter et al. (2012a) investigate whether one can actively enhance the beneficial effect of sleep on creativity by covertly reactivating the creativity task during sleep. Individuals’ creative performance was compared after three different conditions: sleep-with-conditioned-odor; sleep-with-control- odor; or sleep-with-no-odor. In the evening prior to sleep, all participants were presented with a problem that required a creative solution. In the two odor conditions, a hidden scent-diffuser spread an odor while the problem was presented. In the sleep-with-conditioned-odor condition, task reactivation during sleep was induced by means of the odor that was also presented while participants were informed about the problem. In the sleep-with-control-odor condition, participants were exposed to a different odor during sleep than the one diffused during problem presentation. In the no odor condition, no odor was presented. After a night of sleep with the conditioned odor, participants were found to be more creative and better able to select their most creative idea than participants who had been exposed to a control odor or no odor while sleeping. Task reactivation during sleep seems to actively trigger creativity-related processes during sleep. These findings give a first indication that one can manipulate unconscious thought processes and, thereby, facilitate creative performance.

The idea that unconscious processes work on a problem in the absence of conscious guidance has been described by many great artists and thinkers, and the above mentioned findings provide first scientific evidence for the idea that a period of incubation benefits from unconscious processes. This may be related to the fact that unconscious thought organizes information. Representations become better organized and more polarized, and memory becomes more gist-based. Moreover, unconscious thought theory postulates that unconscious thought leads to a process of weighting whereby the importance of information is assessed. However, this idea awaits further study, as it was supported in some experiments (Bos et al., 2011; Usher et al., 2011), but not in others (Ashby et al., 2011; Pachur and Forrer, 2013). These findings may suggest that unconscious thought is a process whereby disorganized information becomes more and more organized until some kind of equilibrium is reached, and the conclusions can be transferred to consciousness. Recently, the first neuroscientific evidence into unconscious thought was provided. As in earlier studies on unconscious thought and decision-making, Creswell et al. (2013) showed that unconscious thinkers made better decisions than conscious thinkers and than immediate decision makers. Moreover, their fMRI data demonstrated that participants who thought unconsciously while doing a distraction task showed more activity in the right DLPFC and left intermediate visual cortex than participants who merely performed the same distraction task. These areas were already involved in the initial encoding of the information in the first place, and the authors proposed a “neural reactivation account” for unconscious thought, indeed demonstrating unconscious processing to continue after encoding. Importantly, neural reactivation in the right DLPFC and left intermediate visual cortex was predictive of decision quality of unconscious thinkers. Further neuroscientific research on creativity and incubation should investigate brain activity during incubation to shed further light on the underlying cognitive mechanisms of incubation effects.

To conclude, several studies suggest that it is not merely the absence of conscious thought that drives creativity, but that during an incubation period unconscious processes can contribute to creative thinking. Often, it takes time to come up with creative ideas and solutions. It is reasonable to assume that most thought processes underlying creative thought are neither fully conscious nor fully unconscious. Instead, prolonged creative thought processes may have both conscious and unconscious elements, and conscious and unconscious thought may alternate. You think about a problem consciously, you get stuck and perform another task, you think some more, you sleep on it for a while, you then think a bit more after you’ve encountered relevant new information, et cetera.

## Practical Applications and Future Research

Previous studies have shown that creativity training can enhance everyday creative performance (e.g., Scott et al., 2004), and many tactics have been identified to facilitate creative thinking skills, such as set-shifting, questioning assumptions, and using analogies (i.e., finding correspondence of inner relationship or function between different concepts). The application of unconscious processes, however, has not been systematically introduced to educational, innovation and business contexts. By demonstrating that unconscious processes can be important for creativity, the current findings may encourage practitioners to use unconscious processes in order to enhance creative thinking. Applying unconscious processes could, for example, entail that people set a goal to find creative solutions for a problem before they are distracted from the problem by doing something different. What people do in the meantime should be chosen carefully. Sio and Ormerod’s (2009) meta-analytic review revealed that the benefits of an incubation period are greater when participants are occupied by an undemanding task than when they engage in a demanding task or no task at all. Moreover, Gilhooly et al. (2013) found that spatial incubation benefited verbal-rated creativity, and verbal incubation benefited spatial-rated creativity but not vice versa. Therefore, when stuck on a creative task, during an incubation period one should do something undemanding that is very different from the main task, before returning to it.

Although unconscious processes can be a powerful source to facilitate creativity, only engage in daydreaming or sleeping to produce groundbreaking discoveries or great artistic creations will not do the trick. A plethora of raw materials has to be available to be connected and one has to be able to focus on some options out of an array of options. In this sense, conscious processing is needed to establish a knowledge base, to know what problems to tackle, and to verify and implement new ideas. Future research may investigate what combination of conscious and unconscious processes is most fruitful for creativity. One could think about the order of the two processes (e.g., a period of task-related conscious thought that is followed by a period during which one refrains from task-related conscious thought, or repeatedly switch between the two modes of thought), and the optimal duration of each of the two processes. People are likely to benefit more from an incubation period when they get stuck and, therefore, one can assume that a relatively long period of conscious thought should be preferred above a short period of conscious thought. Also for unconscious processes the duration of the incubation period seems to be of importance. In a recent study from Yang et al. (2012), 3 min (as compared to 1 min and 5 min) seemed to be the optimal duration of unconscious thought. However, it is likely that 3 min of incubation is not the most appropriate duration for all creativity tasks. It can be assumed that the optimal duration is contingent on the task (Weisberg, 1999). Besides exploring the optimal duration of unconscious processes as a function of task characteristics and the optimal combination of conscious and unconscious processes, future research could also focus on the similarities and disparities between the different unconscious processes (i.e., incubation, unconscious thought, mind-wandering and sleep) and could investigate which process is most beneficial for creativity and for the distinct mental processes underlying creative thought (Baer, 1998). Finally, future research may study potential moderators, for example, whether experts and people with ample prior knowledge exhibit a different pattern of creative performance as a result of unconscious and conscious processes.

The present article aimed to provide an overview of the domain of incubation and creativity, and to shed more light on the causes of incubation effects. Research on incubation, mind wandering, and sleep was presented and discussed, and it was investigated whether people can think unconsciously and whether unconscious processes can contribute to creativity. The current findings provide first empirical support for the idea that during an incubation period unconscious processes contribute to creative thinking, and that it is not merely the absence of conscious thought that drives incubation effects. We hope that the current article inspires researchers to further tackle the unconscious foundations of creativity. This will not only increase our theoretical knowledge on the role of unconscious processes in creativity, but will also offer valuable insights for practical implication. Understanding and facilitating creativity is important, as the ability to think creatively plays an important role in many areas of our life, such as education, arts, sciences, and the economic sector.

## Conflict of interest statement

The authors declare that the research was conducted in the absence of any commercial or financial relationships that could be construed as a potential conflict of interest.

## Footnotes

^1^Note that the section below greatly overlaps with Dijksterhuis (submitted for publication).
